# A Multidisciplinary Approach for Improving Quality of Life and Self-Management in Diabetic Kidney Disease: A Crossover Study

**DOI:** 10.3390/jcm9072160

**Published:** 2020-07-08

**Authors:** Nancy Helou, Dominique Talhouedec, Maya Zumstein-Shaha, Anne Zanchi

**Affiliations:** 1 School of Health Sciences (HESAV), HES-SO University of Applied Sciences and Arts Western Switzerland, 1011 Lausanne, Switzerland; 2 Clinique de La Source, 1004 Lausanne, Switzerland; D.Talhouedec@lasource.ch; 3 Department of Health, Bern University of Applied Sciences, 3010 Bern, Switzerland; maya.zumsteinshaha@bfh.ch; 4 Service of Nephrology and Hypertension, Service of Endocrinology, Diabetes and Metabolism, Department of Medicine, Lausanne University Hospital and University of Lausanne, 1011 Lausanne, Switzerland; Anne.Zanchi@chuv.ch

**Keywords:** self-management support, self-management interventions, multidisciplinary care, interdisciplinary studies, diabetic nephropathy, randomized crossover

## Abstract

Individuals with diabetic kidney disease are at high risk of complications and challenged to self-manage. Previous research suggested that multidisciplinary approaches would improve health outcomes. This study investigated the effect of a multidisciplinary self-management approach of diabetic kidney disease on quality of life, and self-management, glycemic control, and renal function. A uniform balanced crossover design was used because it attains a high level of statistical power with a lower sample size. A total of 32 participants (aged 67.8 ± 10.8) were randomized into four study arms. In differing sequences, each participant was treated twice with three months of usual care alternated with three months of multidisciplinary management. The intervention improved the present dimension of quality of life demonstrating higher mean rank as compared to usual care (52.49 vs. 41.01; *p* = 0.026, 95% CI) and three self-care activities, general diet habits, diabetes diet habits, and blood sugar testing (respectively: 55.43 vs. 38.31; *p* = 0.002, 56.84 vs. 37.02; *p* = 0.000, 53.84 vs. 39.77; *p* = 0.008; 95% CI). Antihypertensive medication engagement was high across the study period (Mean = 95.38%, Min = 69%, Max = 100%). Glycemic control and renal function indicators were similar for the intervention and the usual care. Studies are needed to determine how the new recommended therapies for diabetic kidney disease such as SGLT2 inhibitors and GLP-1 receptor agonists impact on self-management and quality of life.

## 1. Introduction

Worldwide healthcare systems are facing the challenges of the high prevalence [1] and the progressive increase [2] of Diabetic Kidney Disease (DKD). 

DKD is a micro-vascular complication of diabetes that induces a progressive decline in renal function, over five stages, leading to kidney failure [3]. It remains the leading cause of end-stage kidney disease [4] and highly increases the risk of cardiovascular complications [5]. DKD treatment aims at slowing the progression of the kidney disease, preventing cardiovascular events, and improving Quality of Life (QoL) [3,6], especially since the QoL of people with DKD was shown to decrease progressively in parallel to kidney function decline [7]. Individuals with DKD are challenged by the complexity of the expected daily self-management related to diabetes and other comorbidities like hypertension [6,8,9]. These self-care activities include blood glucose monitoring, nutrition adjustment, physical activity, medication engagement, detection and symptoms management, complications risk reduction, and psychosocial adjustment to treatment [10]. 

The multidisciplinary management of DKD is one way to help individuals overcome self-management challenges, achieve treatment goals, and improve health outcomes [11], especially when consolidated by health education tailored to comorbidities, and addressing fear and coping mechanisms [12].

A meta-analysis of multidisciplinary management of DKD included three randomized controlled trials, and demonstrated a significant improvement in glycated hemoglobin (HbA1c) in multidisciplinary management as compared to standard Usual Care (UC). The results did not show a difference related to other clinical outcomes. Only one included study measured person-reported outcomes and demonstrated an improvement in QoL, self-care activities, and level of knowledge on diabetes. These studies were not based on a nursing theory, and did not include a nursing central role or an individualization of care based on participants’ needs with personalized goal setting [13]. 

In one qualitative study, participants with chronic kidney disease (CKD) expressed needs of guidance and support for achieving self-management [14]. Another study highlighted the importance of considering individuals’ illness perception along with stressors, and expectations related to the disease, oneself, social interactions, and dependency on others [15]. Individuals with CKD seem to have a diverse understanding of the disease and its trajectory but voice a need for information and psychosocial support [16]. Nurses are best fit to answer these expressed needs.

Multidisciplinary self-management support for DKD, which is based on a nursing theory, is understudied. Nurses’ role in multidisciplinary care was described in five main interventions: (1) helping individuals in developing self-care abilities, (2) guiding individuals in symptom monitoring, problem-solving techniques, and establishment of priority health goals, (3) providing a follow-up in different settings and using reminders, (4) monitoring progress towards set goals, and (5) coordinating care. The interventions focused on self-management development for achieving treatment targets. 

Therefore, the Self-Care Deficit Nursing Theory (SCDNT) was adopted to guide this study and the nursing practice within the multidisciplinary program. The SCDNT stipulates that actual, or potential, deficits are established when individuals are unable to meet their self-care demands. Nursing, a helping and health regulatory system, intervenes to support individuals in overcoming these self-care deficits. The nurse delivers deliberate care in coordination with the individual who carries own self-care [17]. A nursing theory-based approach applied to the multidisciplinary management of DKD, in ambulatory services, could improve health outcomes.

The primary aim of this study was to determine the effect of a Multidisciplinary Self- Management Support Program (MSMP) on QoL of adults with DKD as compared to UC. The secondary aim was to determine the effect of the MSMP on self-care activities, glycemic control, and renal function of adults with DKD as compared to UC.

## 2. Materials and Methods

### 2.1. Study Design

A uniform and strongly balanced crossover design was used to determine the effectiveness of MSMP as compared to UC in individuals with DKD. This crossover design represents the ideal crossover that is able to overcome the statistical bias of the carry-over effect [18]. This design is uniform within sequences and within periods: each treatment appears the same number of times within each sequence, and the same number of times within each period. It is balanced with respect to carryover effects, because each treatment precedes every other treatment, including itself, the same number of times.

DKD represents approximately 25–30% of the population with diabetes, limiting the possibility for large sample recruitment. This crossover design is recommended for an efficient comparison of treatments when recruiting a small group of participants in order to attain the same level of statistical power, or precision as a randomized controlled trial. It requires a sample size that is ¼ of the randomized controlled trial sample size, because the within-patient variances are one-fourth that of the inter-patient variances [18].

This can be used for comparing treatments of chronic conditions when the treatment aims at improving quality of life and preventing complications. In crossover design, each participant receives the treatment and serve as his or her own control, thus limiting the mixed effects of the heterogeneity in populations with different comorbidities. The focus of a uniform balanced crossover design is to compare the participants’ responses to two different treatments. This design does not allow for a comparison of the participants within differences in the values of variables over time [18].

UC was considered as one of the treatment options of this crossover, therefore, a washout period was not included in the study. Introducing a washout period would have deprived participants of UC, and prevented any contact between participants and their nephrologists, or diabetologists. Given the chronicity of DKD, keeping participants from standard treatment would have been unethical. Our decision is in line with a recent publication on the crossover design [19].

The study was carried out at the ambulatory services of a university hospital and in private practice. Participants were enrolled in the study for 12 months and were allocated to one of the four sequences of the crossover. They received the UC twice, each time over three months, alternating with twice the MSMP, also each time over three months, in a specific sequence depending on their allocation. All participants crossed over from period 1 to 2, then to 3, and finally to 4 (Table 1 black arrow). With each crossover, the UC or MSMP set of interventions was restarted.

The canton of Vaud ethics committee approved the study protocol 44/13 on 10 July 2013 and its amendment on 3 March 2014. The protocol is accessible at https://bmcnephrol.biomedcentral.com/articles/10.1186/s12882-016-0279.

### 2.2. Study Participants

Nephrologists of the Lausanne university hospital, and diabetologists in private practice recruited participants who were 18 years or older, with a clinical diagnosis of diabetes, and kidney disease, with no cognitive deficit, not on dialysis or with a terminal illness. Kidney disease was confirmed by an estimated glomerular filtration rate (eGFR) < 60 mL/min calculated using the CKD Epidemiology Collaboration (CKD_EPI) formula, or an Albumin/Creatinine ratio (Alb/Cr) ≥30 mg/mmol.

All participants signed an informed consent and were free to withdraw from the study at any time, with no prejudice to their care.

### 2.3. Randomization and Masking

Physicians (nephrologists and diabetologists) recruited the participants and were not involved in randomization. A departmental research nurse, who was not involved in the study, allocated the participants to one of the four study sequences using a computerized random number generator. To ensure assessor blinding, an independent private laboratory blinded to the study protocol and participants’ allocation, carried the analyses of the blood and urine sample, and entered the study clinical data. A computer assistant blinded to treatment allocation was responsible for overseeing the data entry of the self-administered questionnaires. Medication monitoring was recorded daily by the Medication Events Monitoring System (MEMS) over 12 months. An independent statistician blinded to the allocation sequences carried out data analysis. Pre-to-post-test outcome comparisons were done by treatment condition, regardless of the sequence of treatment received, to overcome any influence of unmasking of the intervention to participants (Table 1 grey arrows).

### 2.4. Intervention

The study team and the university hospital nephrologists discussed, amended and approved the intervention, and its documentation materials were pilot tested on two volunteers before the start of the study.

The UC consisted of a follow-up by the general practitioner, nephrologist, and/or diabetologist, including a visit to the nephrologist and the diabetologist once every 3 months.

The MSMP consisted of the same follow-up by the general practitioner, nephrologist and/or diabetologist, but added care was provided by an Advanced Practice Nurse (APN), a nurse specialized in diabetes care and a clinical dietician. The APN ensured referrals to physiotherapists, pharmacists, social workers and other healthcare professionals, when needed.

The MSMP alternated nursing and dietary care with the usual nephrology and diabetology consultations to ensure a direct or telephone contact every two weeks with a healthcare professional. In each MSMP period, participants received two dietary consultations, three nursing consultations at their home or at the ambulatory clinic, and two nursing telephone follow-ups. The description of one of the MSMP sequence is shown in Table 2. Each nursing and dietary consultation lasted for one hour, except the first nursing consultations of each MSMP, which lasted one and a half hours.

The APN was responsible for ensuring evidenced-based nursing, managing the MSMP, and coordinating care between healthcare professionals.

The nursing intervention was structured and based on the SCDNT theory. It was built using specific nursing assessment, follow-up documentations, and educational materials that were adapted to the purpose of the study.

The diabetes specialized nurse conducted a comprehensive initial clinical and psychosocial assessment of the participant, and an evaluation of medication safety. She assisted the participant in setting a priority treatment goal and signing a self-management contract for achieving the goal. She developed a collaborative care plan and delivered nursing interventions to help the participant to meet the set goal. She guided participants in symptom monitoring, and problem-solving techniques. She helped participants develop their self-care abilities, identifying and using their resources, engaging in medication taking, and following an exercise regimen (walking at least 90 min per week) and dietary recommendations. She monitored participants’ progress towards achieving set goals. She provided psychosocial support and teaching on diabetes and kidney protection.

The dietician adopted a self-management approach and set an individualized dietary plan.

To ensure fidelity to the intervention, the APN monitored the scheduled appointments and the self-management contract between the diabetes nurse and the participants, describing the set objectives and the plan of care, along with the follow-up documentations.

### 2.5. Outcome Measures

Data were collected at baseline, and after each follow-up period of 3 months, for all outcome measurements between 1 April 2014 and 20 January 2016.

QoL, the primary outcome of the study, was assessed using the Audit of Diabetes-Dependent QoL (ADDQoL) measure, a self-administered questionnaire of 19 items with a weighted score ranging from −9 to 9. It covers three separate dimensions of QoL: Present QoL, Impact of diabetes on QoL, and Impact of diabetes on life domains including social life, physical health, self-confidence, motivation, feelings about the future, dependency on others, and living conditions (α = 0.947) [20].

Self-care activities were evaluated using the self-reported questionnaire Revised Summary of Diabetes Self-Care Activities (R-SDSCA), and electronic monitoring of anti-hypertensive medication taking. The R-SDSCA is a self-administered questionnaire of 10 items covering independent activity domains namely dietary habits, physical activity, blood glucose monitoring, foot care, and smoking (mean r = 0.47; except for diet r = 0.40) [21].

The engagement in anti-hypertensive medication taking was measured using electronic monitoring MEMS [22]. Medication monitoring was recorded daily by the MEMS over 12 months. An average three-month engagement percent was computed for every UC and MSMP period.

Glycemic control was evaluated through HbA1c measurement and renal function through the measurement of serum creatinine, urinary Alb/Cr and eGFR using CKD_EPI formula.

The number of times that participants used help from the MSMP team, outside the defined frequency, was calculated.

### 2.6. Statistical Analysis and Data Management

Targeted ample size was calculated based on the primary outcome QoL. Considering the crossover design, 40 participants were required to detect a 20% clinically significant absolute difference [23] on the ADDQoL for diabetes complications [24], at an α of 0.05, and a power of 0.08, accounting for a 20% expected drop-out rate.

The data were analyzed using SPSS for Windows version 22. *p*-values < 0.05 were considered statistically significant.

Crossover designs have a paired nature, with each participant serving as their own control. Therefore, we reported results using within-individual-treatment differences accounting for point estimates (mean differences), and precision estimate (SD, min and max), based on reporting recommendations for the results of crossover designs [19]. Differences in variables were calculated from timepoints to baseline, for each sequence, to prohibit extreme values from playing a pivotal role in statistical analysis. This pre-to-post-test outcome comparison was done by treatment condition, regardless of the sequence of treatment received (Table 1 grey arrows). The analysis followed the intent-to-treat principle.

The study was registered at ClinicalTrials.gov NCT01967901, on 18 March 2013.

The deidentified participants’ datasets used and analyzed during this study are available from the corresponding author on reasonable request, with a signed data access agreement.

The majority of our dependent variables were not normally distributed. Consequently, the Mann–Whitney-U test, a non-specific statistical test, was used to look for significant overall differences between the intervention and the control, on the dependent variables, comparing mean ranks rather than medians.

## 3. Results

A total of 84 individuals were found to be eligible for recruitment. A total of 32 (mean age 67.8 ± 10.8; 90.6% men) agreed to participate between 1 April 2014 and 20 January 2015. Participants had, on average, 3.5 comorbidities (Table 3).

**Figure 1:**
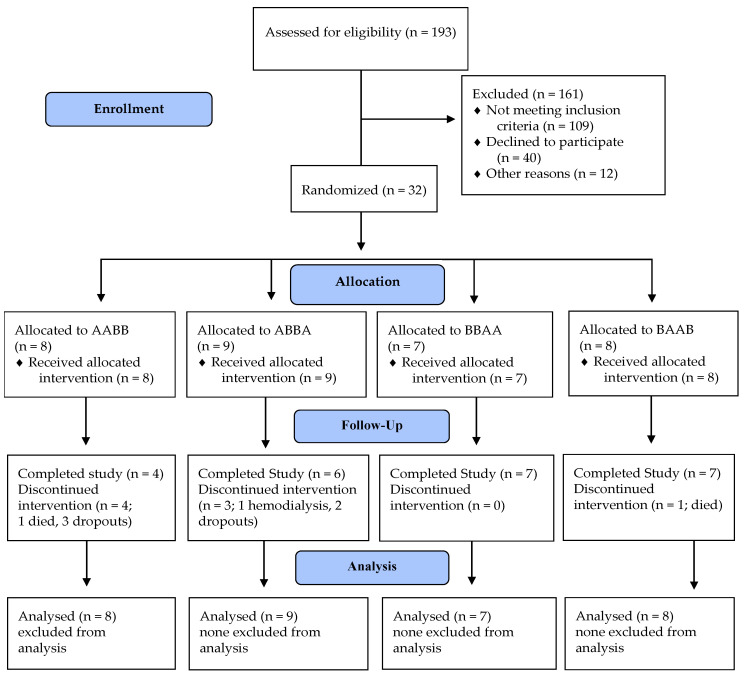
Study profile.

Five participants withdrew from the study. These withdrawals occurred in the two sequences which did not start with the MSMP directly at enrollment. One participant with stage 4 DKD was excluded from the study because his renal function declined, and he started hemodialysis. Two participants passed away during the study (Figure 1).

MSMP was found to improve the general QoL of individuals with DKD as compared to UC, with a highest significant mean rank (52.49 vs. 41.01; *p* = 0.026, 95% confidence interval), considering a 20% improvement as a clinically significant absolute difference [23] (Table 4). However, the UC and the MSMP showed no difference in relation to the impact of diabetes on QoL and on life domains. Three self-care activities, which were at the heart of our intervention, demonstrated significant differences between MSMP and UC. General diet habits, diabetes diet habits and blood sugar testing all showed significant improvement for MSMP as compared to UC (respective ranks 55.43 vs. 38.31; *p* = 0.002, 56.84 vs. 37.02; *p* = 0.000, and 53.84 vs. 39.77; *p* = 0.008; 95% confidence interval).

The antihypertensive medication monitoring results did not differ between MSMP and UC (Table 4). However, the medication monitoring overall percentage mean was high across UC and MSMP, for all participants who used it, over 12 months of the study duration (n = 21, Mean = 95.38%, SD = 7.29, Min = 69%, Max = 100%).

The results did not demonstrate significant differences between MSMP and UC in the clinical indicators related to glycemic control and renal function (Table 4). Absolute values are presented in Supplementary Materials Tables S1–S12.

All participants who completed the study received, in each MSMP period, the full number of consultations as per protocol. Eleven participants needed supplementary consultations, which mainly consisted of nursing telephone calls.

## 4. Discussion

Participants were predominantly men with an age range of 50–91 years (Table 3); thus, our results can mainly be generalized to men. Studies on the multidisciplinary management of DKD were conducted in different sociocultural environments. They reported diverse sex percentage and an age range of 44–74.6% [25,26,27,28,29]. One report showed > 50% higher diabetes prevalence in men [30,31]. Age and sex differences in DKD might be affected by diabetes prevalence, genetic predispositions, sociocultural, and environmental factors. Our intervention was based on the SCDNT, which aims to improve QoL by helping people to gain knowledge on self-management [17]. The results showed a significant increase in general QoL and three of their self-care activities, which is in line with a previous study results [29]. This new approach generated an additional cost of 1200 per patient, extrapolating an annual additional cost of 16–17 million to cover the national DKD population.

The findings are consistent with the SCDNT theoretical assumptions and basis. The nursing theory-based intervention did contribute to the short-term outcomes, namely self-care activities, which are central to the theory and expected to mediate long-term outcomes like the stability of the kidney function. The MSMP was built, as a whole, to promote participants’ capabilities in carrying out their own self-management. Puzzling out the contribution of each self-management activity to the clinical results is not possible at this time.

To our knowledge, our study is the first to measure medication engagement and use of MEMS in DKD. The results revealed a high engagement (over 12 months). In the literature, the electronic monitoring of medication in diabetes was variable, ranging from 53 to 98% [32]. We blinded participants to the daily reading of the MEMS, which was used as a self-dispensing medication container over a long period of 12 months. Consequently, the higher percentage of engagement cannot be related to the use of electronic monitoring.

Mean HbA1c at baseline was 7.7%, which represented a reasonable clinical goal given the higher risk of hypoglycemia in DKD; therefore, and despite a significant increase in blood glucose testing, the results could not demonstrate a further improvement in glycemic control with the multidisciplinary management, in contrast to the meta-analysis that pooled the data of three studies [13]. Two of the studies included in the meta-analysis found no effect of multidisciplinary management on HbA1c at three [29] and 12 months [27], while the third study demonstrated significantly lower HbA1c at 24 months [25].

The present study mean baseline eGFR represented moderate kidney damage (stage 3) congruent with similar studies [25,26,27,28,29]. The results were similar to those of the meta-analysis that could not find evidence supporting multidisciplinary management in delaying the progression of DKD [13], despite the high antihypertensive medication engagement. The actual slowing of the renal function decline rate may become evident after 24 months [33]. Nevertheless, addressing current clinical status [34] and focusing on QoL and daily self-management is central to individuals with DKD. Thus, they would be supported in coping with uncertainty. As a result, their physical and emotional wellbeing would be improved. Only two participants were on GLP-1 receptor agonists and none were on SGLT2 inhibitors because these medications had only just arrived on the Swiss market at the beginning of the study. SGLT2 inhibitors have been shown to reduce the risk of renal disease progression in diabetes [35]. The use of these two medications has recently been recommended [36] and their new combination therapy could be promising for improving blood pressure, glycemic control, renal function [37,38] and QoL [39].

A major limitation of the study can be attributed to the non-attainment of the anticipated recruitment of 40 participants. Recruitment was extended for an additional six-month period and was expanded to diabetologists, and nephrologists of the private sector near the participating hospital. However, more than half of the eligible participants were already involved in other studies.

Two study sequences, starting with UC at enrollment, witnessed participants’ withdrawal. Dropouts did not occur in the two sequences that started with MSMP at enrollment. The retention of participants in the study could have been improved if participants starting with UC were reminded regularly of the study plan. One can assume that, with time, participants were facing the burden of the advancing disease and were less able to invest in research.

With the recent evidence that SGLT2 inhibitors and GLP1-R agonists provide cardio-renal protection in high-risk individuals with type 2 diabetes, their use should now be considered as a standard of care. Studies are required to determine the effect of GLP-1 receptor agonists’ and SGLT2 inhibitors’ use in multidisciplinary self-management support of DKD and QoL. Further research is needed to compare multidisciplinary self-management support to other self-management approaches like the Stanford approach, which relies on peer support [40], or other care delivery modalities like web-based interventions and applications.

## 5. Conclusions

This study was unique in integrating a nursing theory-based intervention within a multidisciplinary management approach. MSMP was a complex intervention aiming to promote self-management. The general QoL and self-management activities of individuals with DKD were improved. The MSMP supported the importance of nursing theory-guided practice and the significant role of specialized nurses as part of multidisciplinary teams. It highlighted the advanced role for nurses in promoting chronic disease self-management. However, multidisciplinary approaches and self-management support should start before or at the early stages of renal disease or albuminuria. A combination of new therapies, namely SGLT2 inhibitors and GLP-1 receptor agonists, may positively contribute to individuals’ self-management abilities and glycemic control, leading to an improvement in renal outcomes, DKD experience and thus QoL. Research should also address the direct effect of the combination of these new therapies on QoL, in individuals with established or advanced DKD.

## Figures and Tables

**Table 1:** Crossover plan of participants enrolled in the study.

	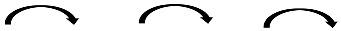			
**Design**	**Period 1** **0–3 Months**	**Period 2** **3–6 Months**	**Period 3** **6–9 Months**	**Period 4** **9–12 Months**
**Sequence ABBA (n = 10)**	UC	MSMP	MSMP	UC
**Sequence BAAB (n = 10)**	MSMP	UC	UC	MSMP
**Sequence AABB (n = 10)**	UC	UC	MSMP	MSMP
**Sequence BBAA (n = 10)**	MSMP	MSMP	UC	UC
	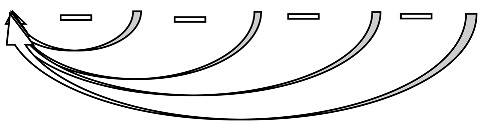			

**Table 2:** The schedule, description, and outcome measurement of the multidisciplinary self-management program (MSMP) sequence-BAAB for patients with diabetic kidney disease (DKD).

			A Multidisciplinary Self-Management Program Sequence BAAB								
			Weeks 1 and 41	Weeks 3 and 43	Weeks 5 and 45	Weeks 7 and 47	Weeks 9 and 49	Weeks 11 and 51	Weeks 12 and 52	Weeks 13, 27, 28, 30 and 40, 52–54	Week 52
	**Assessment and/or Intervention**	Medical visit and Screening	Nurse Home Visit	Nurse Telephone follow-up	Dietician Clinic Visit	Nurse Home Visit	Dietician Clinic Visit	Nurse Telephone Follow-up	Nurse Clinic Visit	Medical Visit nephrologist or diabetologist	End of the study
**Enrollme-nt**	Inclusion/Exclusion criteria	X									
	Information form	X									
	Randomization	X									
	Informed consent form	X									
**Treatment**	1. Comprehensive initial assessment and evaluation of patients’ self-care deficits		X							Medical follow-up	
	Current Medications		X								
	Priority setting-one goal and contract signing		X								
	2. Teaching and Training on self-care		X			X					
	Education on DKD					X					
	Education on the risk of hypoglycemia						X				
	3. Counseling on self-care development					X	X				
	4. Guiding and support			X		X	X				
	5. Coordination of Care		X	X	X	X	X	X	X		
	6. Follow-up and proactive monitoring			X		X		X			
	Dietary plan and counseling				X				X		
**Outcomes’ measurements**	Demographics		X								
	Self-management activities		X						X		X
	Medication adherence		X						X		X
	Quality of life		X						X		X
	Serum cr, eGFR, urinary albumin/cr ratio		X						X		X
	HbA1c		X						X		X
	Resource utilization		X						X		X

**Table 3:** Characteristics of Study Participants (n = 32).

**Clinical Characteristics**		**Range**	**Mean (± SD)/Median**
Age (years)		50–91	67.8 (±10.8)/67
HbA1c (%)		5.7–10.5	7.7 (±1.3)/7.45
eGFR (mL/min/ 1.73 m^2^)		15–108	41.3 (±21.5)/35
**Sociodemographic Characteristics**		**Frequency**	**%**
Age Group	50–64	14	43.8
	65–80	13	40.6
	≥80	5	15.6
Sex	Men	29	90.6
	Women	3	9.4
Marital Status	Married/ Cohabitating	21	65.6
	Divorced/Living alone	11	34.4
Professional Status	Retired	18	56.3
	Independent/Employed	10	31.2
	Medical cessation of work	4	12.5
Educational Level	Academic	11	34.4
	Short-cycle/Post-secondary	12	37.5
	Primary	9	28.1
Comorbidities	Cardiovascular disease	15	47
	Dyslipidemia	20	63
	Neuropathy	14	44
	Retinopathy	14	44
	Hypertension	30	94
	Peripheral vascular disease	8	25
	Gout	3	9
	Sleep apnea	7	22
Medications	Insulin	22	69
	ACE inhibitor or ARB	27	84
	GLP-1Ra	2	6
	SGLT2i	0	0

**Table 4:** Changes in quality of life (QoL), Self-Management, Glycemic Control, and Renal Function between MSMP and usual care (UC).

Variable	Measure	Pre-Post Difference	n	Mean Rank	Rank Sum	Mean	SD	n	Mean Rank	Rank Sum	Mean	SD	M-U	Sig.
			UC					MSMP						
***Quality of Life***	ADDQoL	Present QoL	48	41.01	1968.5	−0.19	0.87	44	52.49	2309.5	0.18	1.06	792.5	0.026 *
		DM Impact on QoL	48	47.24	2267.5	0.06	1.19	44	45.69	2010.5	−0.05	0.96	1021	0.763
		DM Impact on Domains	48	43.03	2065.5	−0.11	0.95	44	50.28	2212.5	−0.06	1.44	889.5	0.193
***Self-Management***	*R-SDSCA*	General Diet Habits	48	38.31	1839	−0.46	1.94	44	55.43	2439	0.93	1.66	663.0	0.002 *
		DM Specific Diet Habits	48	37.02	1777	−0.19	1.06	44	56.84	2501	0.82	1.85	601.0	0.000 *
		Exercise Habits	48	45.21	2170	0.30	2.66	44	47.91	2108	0.47	2.38	994.0	0.626
		Blood Sugar Testing	48	39.77	1909	−0.58	2.54	44	53.84	2369	0.80	2.22	733.0	0.008 *
		Foot Care	48	43.34	2080.5	−0.14	1.88	44	49.94	2197.5	0.25	1.65	904.5	0.214
		% of Smoking Status	48	47.90	2299	0.04	0.20	44	44.98	1979	−0.02	0.15	989.0	0.089
		No· of cigarettes/ day	9	10.22	92	−0.44	4.10	9	8.78	79	−1.22	6.22	34.00	0.558
	MEMS	Anti-HTN Medication	26	21.79	566.5	−3.08	12.1	22	27.71	609.5	−1.32	6.44	215.5	0.140
***Glycemic Control***	%	HbA1c	56	56.51	3164.5	0.06	0.64	50	50.13	2506.5	−0.17	0.87	1232	0.285
***Renal Function***	µmol/L	Serum Creatinine	55	52.62	2894	−3.44	32.3	51	54.45	2777	1.47	28.8	1354	0.759
	mL/min	eGFR (CKD_EPI)	57	57.75	3291.5	1.51	12.4	51	50.87	2594.5	−0.20	9.48	1269	0.254
	mg/mmol	Urine Alb/Cr	46	49.35	2270	9.89	103	46	43.65	2008	−2.60	103	927.0	0.306

Anti-HTN= Antihypertensive; DM = Diabetes Mellitus; Exercise Habits = frequency of physical activity /week; M-U = Mann–Whitney U; * Significant *p*-values; Intent to treat data

## Supplementary Materials

The following are available online at https://www.mdpi.com/2077-0383/9/7/2160/s1, Table S1: Present QoL Absolute Values by Sequence, Table S2: Self-Management General Diet Habits Absolute Values by Sequence, Table S3: Self-Management Specific Diet Habits Absolute Values by Sequence, Table S4: Self-Management Blood Sugar Testing Absolute Values by Sequence, Table S5: DM Impact on QoL Values by Sequence, Table S6: DM Impact on Domains Values by Sequence, Table S7: Self-Management Exercise Habits (Frequency of Physical Activity) Values by Sequence, Table S8: Self-Management Foot Care Values by Sequence, Table S9: Glycemic Control Values by Sequence, Table S10: Renal Function Serum Creatinine Values by Sequence, Table S11: Renal Function eGFR (CKD_EPI) Values by Sequence, Table S12: Renal Function Urine Alb/Cr Values by Sequence.
