# Supplementary material for: A Multidisciplinary Approach for Improving Quality of Life and Self-Management in Diabetic Kidney Disease: A Crossover Study
Source: J Clin Med. 2020 Jul 8;9(7):2160. doi: 10.3390/jcm9072160 (PMC7408890; doi:10.3390/jcm9072160)
Supplement: Supplementary file 1 [file jcm-09-02160-s001.pdf]

Supplementary Materials

Table S1: Present QoL Absolute Values by Sequence

Sequence	Time (months) 0	3	6	9	12
	Mean (-3 to +3) and SD				
ABBA (n=9)	Baseline<br>0·11± 0·78	UC<br>-0·25±1·39	MSMP<br>0·14±1·07	MSMP<br>0·67±1·03	UC<br>0·67±0·82
BAAB (n=8)	Baseline<br>0·25±0·89	MSMP<br>1·14±1·07	UC<br>1·20±0·84	UC<br>1·50±0·58	MSMP<br>0·43±1·4
AABB (n=8)	Baseline<br>1·25±0·89	UC<br>0·71±1·11	UC<br>0·67±1·21	MSMP<br>0·50±0·58	MSMP<br>0·50±0·58
BBAA (n=7)	Baseline<br>1·00±1·29	MSMP<br>1·00±1·15	MSMP<br>1·00±1·29	UC<br>0·83±1·33	UC<br>0·43±1·99

Table S2: Self-Management General Diet Habits Absolute Values by Sequence

Sequence	Time (months) 0	3	6	9	12
	Mean (0 to 7 day/ week) and SD				
ABBA (n=9)	Baseline<br>4·22± 2·83	UC<br>1·87±2·37	MSMP<br>4·71±2·39	MSMP<br>5·42±2·73	UC<br>4·92±2·24
BAAB (n=8)	Baseline<br>3·81±2·43	MSMP<br>5·07±1·64	UC<br>4·90±1·52	UC<br>4·75±0·96	MSMP<br>5·29±1·22
AABB (n=8)	Baseline<br>4·81±2·64	MSMP<br>5·14±2·11	UC<br>4·58±2·2	UC<br>4·00±2·68	MSMP<br>5·25±1·26
BBAA (n=7)	Baseline<br>4·64±2·73	MSMP<br>6·28±1·5	MSMP<br>6·36±1·5	UC<br>5·67±1·72	UC<br>5·78±1·63

Table S3: Self-Management Specific Diet Habits Absolute Values by Sequence

Sequence	Time (months) 0	3	6	9	12
	Mean (0 to 7 day/ week) and SD				
ABBA (n=9)	Baseline<br>2·78± 1·52	UC<br>2·81±1·41	MSMP<br>4·78±0·95	MSMP<br>4·83±1·7	UC<br>4·17±1·54
BAAB (n=8)	Baseline<br>3·37±1·22	MSMP<br>4·14±1·21	UC<br>3·70±1·44	UC<br>4·37±0·95	MSMP<br>4·36±0·8
AABB (n=8)	Baseline<br>4·12±1·96	MSMP<br>3·36±0·85	UC<br>3·08±0·1·11	UC<br>3·62±0·85	MSMP<br>3·87±1·97
BBAA (n=7)	Baseline<br>2·50±2·24	MSMP<br>4·36±2·17	MSMP<br>5·28±1·78	UC<br>5·08±1·2	UC<br>4·86±1·25

Table S4: Self-Management Blood Sugar Testing Absolute Values by Sequence

Sequence	Time (months)				
	0	3	6	9	12
	Mean (0 to 7 day/ week) and SD				
ABBA (n=9)	Baseline<br>3·33± 3·11	UC<br>3·62±3·38	MSMP<br>3·64±3·35	MSMP<br>5·92±1·43	UC<br>3·75±2·82
BAAB (n=8)	Baseline<br>3·31±2·11	MSMP<br>4·64±2·93	UC<br>4·10±2·36	UC<br>3·37±3·26	MSMP<br>4·14±3·4
AABB (n=8)	Baseline<br>4·06 ±2·58	MSMP<br>4·71±2·3	UC<br>4·67±2·87	UC<br>5·75±2·5	MSMP<br>6·00±1·15
BBAA (n=7)	Baseline<br>4·86±2·11	MSMP<br>4·71±2·93	MSMP<br>5·28±2·36	UC<br>3·58±3·26	UC<br>3·07±3·4

Table S5: DM Impact on QoL Values by Sequence

Sequence	Time (months)				
	0	3	6	9	12
	Mean (-3 to +3) and SD				
ABBA (n=9)	Baseline<br>-1.44± 1.24	UC<br>-1.38±1.06	MSMP<br>-1.43±1.13	MSMP<br>-1.33±1.21	UC<br>-1.67±0.52
BAAB (n=8)	Baseline<br>-1.87±0.83	MSMP<br>-2.00±0.82	UC<br>-2.40±0.89	UC<br>-1.75±1.5	MSMP<br>-2.29±0.76
AABB (n=8)	Baseline<br>-1.50±1.07	MSMP<br>-1.43±1.51	UC<br>-0.67±1.03	UC<br>-1.50±1.30	MSMP<br>-2.00±1.41
BBAA (n=7)	Baseline<br>-1.43±1.51	MSMP<br>-1.29±1.38	MSMP<br>-1.28±1.11	UC<br>-1.33±1.21	UC<br>-1.14±0.9

Table S6: DM Impact on Domains Values by Sequence

Sequence	Time (months)				
	0	3	6	9	12
	Mean (-9 to +9) and SD				
ABBA (n=9)	Baseline<br>-2.62± 2.2	UC<br>-3.25±1.87	MSMP<br>-2.9±1.54	MSMP<br>-3.87±3.34	UC<br>-3.63±2.67
BAAB (n=8)	Baseline<br>-2.73±2.35	MSMP<br>-2.32±1.75	UC<br>-2.69±1.6	UC<br>-2.81±1.68	MSMP<br>-3.34±2.4
AABB (n=8)	Baseline<br>-2.57±2.78	MSMP<br>-2.11±3.06	UC<br>-2.19±2.85	UC<br>-3.20±3.41	MSMP<br>-3.44±3.03
BBAA (n=7)	Baseline<br>-2.17±2	MSMP<br>-2.31±2.29	MSMP<br>-2.67±2.71	UC<br>-2.81±2.87	UC<br>-3.00±2.87

Table S7: Self-Management Exercise Habits (Frequency of Physical Activity) Values by Sequence

Sequence	Time (months)				
	0	3	6	9	12
	Mean (0 to 7 day/ week) and SD				
ABBA (n=9)	Baseline<br>1.83± 3	UC<br>3.62±2.72	MSMP<br>2.5±2.55	MSMP<br>4.58±1.88	UC<br>4.33±1.66
BAAB (n=8)	Baseline<br>3.00±1.81	MSMP<br>2.64±1.93	UC<br>3.60±2.4	UC<br>4.25±1.85	MSMP<br>3.78±2.12
AABB (n=8)	Baseline<br>4.25±2.95	MSMP<br>5.00±2.84	UC<br>3.67±2.68	UC<br>4.87±3.27	MSMP<br>3.12±2.17
BBAA (n=7)	Baseline<br>2.86±3.25	MSMP<br>4.36±2.32	MSMP<br>3.93±2.59	UC<br>4.17±1.97	UC<br>4.36±2.84

Table S8: Self-Management Foot Care Values by Sequence

Sequence	Time (months)				
	0	3	6	9	12
	Mean (0 to 7 day/ week) and SD				
ABBA (n=9)	Baseline<br>4.83± 2.44	UC<br>3.56±2.65	MSMP<br>4.71±2.61	MSMP<br>5.17±1.75	UC<br>4.41±2.29
BAAB (n=8)	Baseline<br>2.75±2.45	MSMP<br>4.36±1.82	UC<br>4.20±2.2	UC<br>3.12±1.31	MSMP<br>3.14±1.75
AABB (n=8)	Baseline<br>3.06±2.04	MSMP<br>3.57±3.2	UC<br>4.50±3.32	UC<br>3.50±2.2	MSMP<br>4.37±1.8
BBAA (n=7)	Baseline<br>1.50±1.87	MSMP<br>2.14±2.44	MSMP<br>2.21±2.53	UC<br>3.58±3.14	UC<br>3.14±3.09

Table S9: Glycemic Control Values by Sequence

Sequence	Time (months)				
	0	3	6	9	12
	Mean (% HbA1c) and SD				
ABBA (n=9)	Baseline<br>7.47± 0.98	UC<br>7.70 ± 1.02	MSMP<br>7.30 ± 1.15	MSMP<br>7.14± 0.69	UC<br>7.37 ± 0.78
BAAB (n=8)	Baseline<br>8.62 ± 1.67	MSMP<br>8.49 ± 1.56	UC<br>9.12 ± 1.1	UC<br>8.65± 0.66	MSMP<br>8.60 ± 1.19
AABB (n=8)	Baseline<br>7.42 ± 0.96	MSMP<br>7.29 ± 0.84	UC<br>7.24 ± 0.59	UC<br>7.78± 1.32	MSMP<br>8.12 ± 0.35
BBAA (n=7)	Baseline<br>7.17 ± 0.94	MSMP<br>6.74 ± 0.76	MSMP<br>6.76 ± 0.85	UC<br>7.04± 1.44	UC<br>6.77 ± 0.97

Table S10: Renal Function Serum Creatinine Values by Sequence

Sequence	Time (months)<br>0	3	6	9	12
	Mean (µmol/L) and SD				
ABBA (n=9)	Baseline<br>186.00± 78.43	UC<br>180.78±84.06	MSMP<br>212.87±93.40	MSMP<br>182.50±47.53	UC<br>174.83±41.84
BAAB (n=8)	Baseline<br>160.50±60.91	MSMP<br>145.14±49.01	UC<br>155.33±49.25	UC<br>141.57±44.1	MSMP<br>142.28±61.44
AABB (n=8)	Baseline<br>172.25± 61.44	MSMP<br>183.57± 76.50	UC<br>166.62±67.81	UC<br>189.60±79.80	MSMP<br>154.25±36.34
BBAA (n=7)	Baseline<br>164.86± 78.54	MSMP<br>160.71±72.62	MSMP<br>160.14±76.62	UC<br>160.14±80.82	UC<br>162.00±61.26

Table S11: Renal Function eGFR (CKD_EPI) Values by Sequence

Sequence	Time (months)<br>0	3	6	9	12
	Mean (ml/min) and SD				
ABBA (n=9)	Baseline<br>37.00±20.20	UC<br>39.55±21.34	MSMP<br>31.00±15.13	MSMP<br>32.67±10.89	UC<br>33.67±11.96
BAAB (n=8)	Baseline<br>45.50±21.47	MSMP<br>48.71±22.39	UC<br>46.17±24.38	UC<br>50.29±19.16	MSMP<br>53.14±25.32
AABB (n=8)	Baseline<br>37.62±14.80	MSMP<br>43.87±22.39	UC<br>42.25±18.75	UC<br>38.20±20.03	MSMP<br>42.50±12.18
BBAA (n=7)	Baseline<br>46.28± 30.79	MSMP<br>48.00±31.27	MSMP<br>48.57±30.32	UC<br>53.00±33.79	UC<br>42.50±22.58

Table S12: Renal Function Urine Alb/Cr Values by Sequence

Sequence	Time (months)<br>0	3	6	9	12
	Mean (mg/mmol) and SD				
ABBA (n=9)	Baseline<br>77.32±135.21	UC<br>133.56±198.97	MSMP<br>91.40±139.71	MSMP<br>13.74±12.53	UC<br>40.49±21.98
BAAB (n=8)	Baseline<br>96.04±213.12	MSMP<br>84.25±176.21	UC<br>38.07±41.45	UC<br>60.18±89.18	MSMP<br>130.21±263.32
AABB (n=8)	Baseline<br>74.69±93.76	MSMP<br>114.86±122.43	UC<br>49.27±69.13	UC<br>110.58±135.06	MSMP<br>92.08±122.11
BBAA (n=7)	Baseline<br>33.29±31.28	MSMP<br>35.30±43.27	MSMP<br>32.28±38.14	UC<br>44.72±83.82	UC<br>62.06±114.86
